# Cardiac Imaging and Management of Prosthetic Valve Candida Parapsilosis Endocarditis

**DOI:** 10.7759/cureus.16082

**Published:** 2021-07-01

**Authors:** Arjun C Khadilkar, Jesus Diaz Vera, Juan Enciso, Paula Hernandez Burgos

**Affiliations:** 1 Internal Medicine, University of South Florida, Tampa, USA; 2 Cardiology, University of South Florida, Tampa, USA

**Keywords:** candida endocarditis, prosthetic heart valve, fungal, splenic infarcts, transthoracic and transesophageal echocardiography

## Abstract

Fungal infective endocarditis is a rare and serious form of endocarditis associated with severe morbidity and mortality. The greatest propensity for infection can be found in patients with implanted prosthetic valves, implanted cardiac devices, and intravenous drug use. We present a case of a 45-year-old male with a prior bioprosthetic mitral valve who was diagnosed with *Candida parapsilosis* endocarditis. Computed tomography imaging of the abdomen was significant for splenic infarcts, and transesophageal echocardiography demonstrated a 1.23 cm x 0.55 cm lesion and 1.02 cm x 0.545 cm lesion on the bioprosthetic valve. The patient was subsequently treated with Amphotericin B and life-long Fluconazole. This case highlights the imaging findings and treatment of a rare disease process.

## Introduction

Fungal infective endocarditis (IE) is a rare and serious form of IE with mortality rates up to 50% [1]. Typical risk factors include prosthetic valve implantation, cardiac implantation devices, and intravenous drug use [1]. In general, the most common etiologic causes of fungal IE are the Candida and Aspergillus species [1]. A six-year case review examining 12 separate cases of Candida IE found the most common organisms were *Candida parapsilosis* (n = 8, 67%), *Candida glabrata* (n = 3, 25%), and *Candida albicans* (n = 1, 8%) [2]. Current guidelines by the Infectious Diseases Society of America Candidiasis and American Heart Association Endocarditis recommend treatment of Candida IE with either Amphotericin B with or without Flucytosine or high-dose echinocandin therapy, followed by life-long maintenance therapy with an oral azole [2]. However, treatment failure with medical management alone is common because Candida species have adapted survival strategies including the formation of biofilms on native and prosthetic heart valves, which can lead to poor antifungal activity [1]. Therefore, it is imperative that the treatment of native valve endocarditis includes a potential surgical option, especially in patients with prosthetic valves since early intervention has been proven beneficial [1]. Despite combined medical and surgical treatment, fungal IE has a poor overall prognosis, and there is an imperative need for aggressive risk factor management.

We present a case of a 45-year-old male with *Candida parapsilosis* IE in the setting of a bioprosthetic mitral valve and highlight the computed tomography (CT) abdomen and pelvis, transthoracic echocardiography (TTE), and transesophageal echocardiography (TEE) image findings associated with this devastating disease process.

## Case presentation

A 45-year-old male with a history of intravenous drug use, liver cirrhosis secondary to hepatitis C, recent mitral valve endocarditis with a bioprosthetic mitral valve in 2019, gastroesophageal reflux disease (GERD), and pancytopenia presented with four months of progressive abdominal distention. The patient lost his insurance during the coronavirus-19 pandemic and was not following up with routine healthcare. His abdominal distention was causing decreased appetite and early satiety and led to a 40-pound unintentional weight loss. The patient denied associated symptoms of nausea, vomiting, or diarrhea and felt that his symptoms of GERD were well-controlled with daily pantoprazole. In addition, the patient was having intermittent fevers for two weeks and shortness of breath for three days prior to admission.

On admission, the patient was febrile with a temperature of 101 degrees Fahrenheit, normotensive with blood pressure 116/77 millimeters of mercury (mmHg), tachycardia with a heart rate of 114 beats per minute, and saturating 98% on room air. Initial labs are listed in Table 1.

**Table 1:** Patient's labs on presentation

Name of Lab	Patient Value	Normal Range
High-sensitivity C-reactive protein	6.75 mg/dL	0.01-0.05 mg/dL
Aspartate transaminase (AST)	41 U/L	5-34 U/L
Total bilirubin	1.9 mg/dL	0.1-1.2 mg/dL
Sedimentation rate	41 mm/hr	0-15 mm/hr
Troponin	<0.010 ng/mL	0.000-0.028 mg/dL
White blood cell count	5.26 x 10^3^/uL	4.6-10.2 x 10^3^/uL
Hemoglobin	9.7 g/dL	14.1-18.1 g/dL
Platelets	45 x 10^3^/uL	142-424 x 10^3^/uL
Hepatitis C viral load	<10 IU/mL	<10 IU/mL

**Figure 1:**
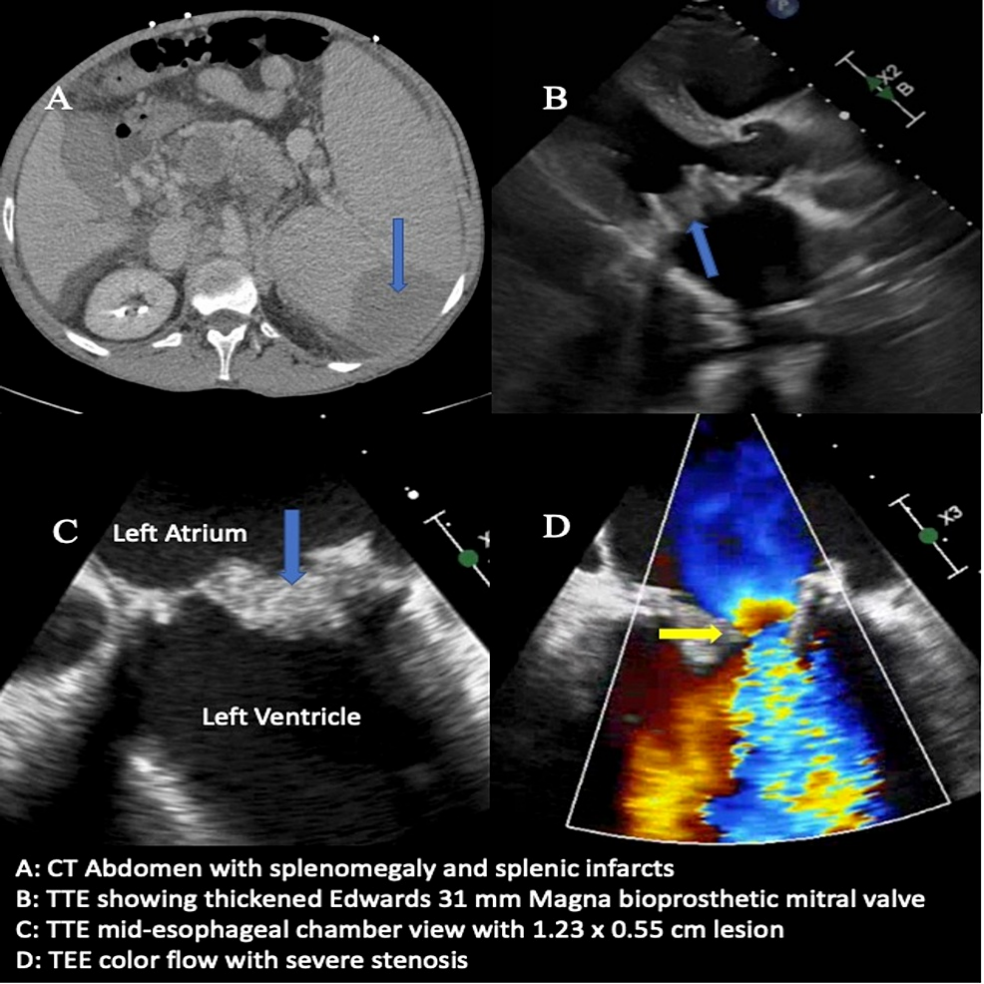
Computed tomography of the abdomen, transthoracic echocardiography, and transesophageal echocardiography findings D: Doppler findings; mitral mean velocity: 2.7 m/sec; mitral mean gradient: 17 mm Hg (severe > 10 mm Hg)

CT abdomen and pelvis were remarkable for splenomegaly with new acute to subacute splenic infarcts, the presence of cirrhosis with moderate volume ascites, and portal enteropathy (Figure 1). Chest x-ray showed worsening bilateral airspace disease with perihilar distribution, and CT angiography of the pulmonary arteries was negative for acute central pulmonary embolus. Blood cultures were ordered, and the patient was started on empiric Cefepime.

**Figure 2:**
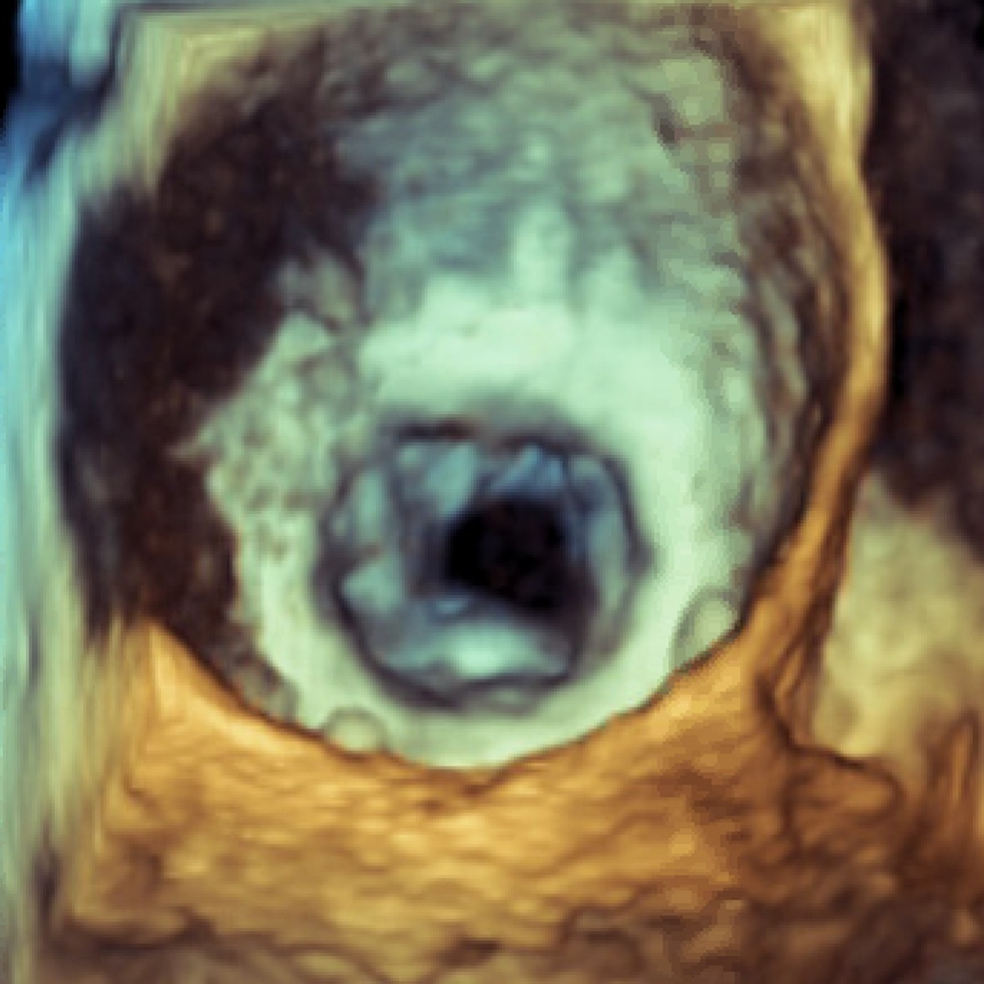
Three-dimensional transesophageal echocardiogram of surgeon's view during diastole Three-dimensional planimeter valve area: 0.77-0.87 cm^2^ (Severe < 1.0 cm^2^).

Thoracentesis was performed to relieve his respiratory symptoms. Approximately 1200 milliliters of cloudy amber fluid was drained out, which had moderate white blood cells without the growth of any organisms. TTE showed a preserved left ventricular ejection fraction of 55%-60% and a 31-millimeter (mm) Edwards bioprosthetic valve with severely thickened leaflets and restricted motion. TTE was unequivocal in completely excluding any vegetation or mass on the bioprosthetic valve. The mean mitral valve gradient was elevated at 28 mm Hg (normal < 13 mm Hg), and the pulmonary artery pressure was 74 mm Hg (normal 18-25 mm Hg) (Figure 1). Initial blood cultures grew in *Candida parapsilosis*, and the patient was started on intravenous (IV) Micafungin 100 mg daily. TEE study performed subsequently during the same anesthesia showed a 1.23 cm x 0.55 cm lesion and 1.02 cm x 0.55 cm lesion on the Edwards 31 mm Magna bioprosthetic mitral valve consistent with vegetation versus thrombus (Figure 2). The patient was deemed not a surgical candidate due to concomitant liver cirrhosis, esophageal varices, ascites, and elevated model for end-stage liver disease (MELD) score of 13-15. He was temporarily treated with IV Amphotericin B 200 mg daily for seven days and was ultimately discharged with six weeks of IV Fluconazole 400 mg daily followed by life-long oral Fluconazole 200 mg daily.

## Discussion

Fungal prosthetic valve IE is a rare presentation of IE affecting people worldwide. In a prior literature review, a total of 152 cases were identified from 1995 to 2000, with intravenous injection drug use as an identified risk factor in only 4.1% of cases [3]. Fungal IE is associated with high incidences of severe morbidity and mortality, ranging from overall morbidity of 67% and six-month mortality risk of 37% [4,5]. The rarity of published reports and the medical guidelines for the appropriate choice and duration of antifungal therapy has been limited. Of those case reports published, medical management with liposomal Amphotericin B (LAmB) 5 mg/kg/day (300 mg/day) and Flucytosine 150 mg/kg/day (9 g/day) as part of an initial treatment after positive blood cultures for Candida is the suggested intervention [6]. The latest Infectious Diseases Society of America (IDSA) from 2016 provides guidelines regarding the treatment of Candida infections with relationship to infected pacemakers, implantable cardiac defibrillators, ventricular assist devices, along with the treatment of native valve and prosthetic valve IE [7]. These guidelines recommend LAmB of 3-5 milligrams (mg) per kilogram (kg), with or without Flucytosine 25 mg/kg four times daily, or high-dose echinocandin therapy (Caspofungin 150 mg daily, Micafungin 150 mg daily, or Anidulafungin 200 mg daily) [7]. In addition, for patients unable to undergo valve replacement, there is a strong recommendation for chronic long-term suppression with Fluconazole 400-800 mg daily [7]. Our patient continues to follow up with cardiology as an outpatient. The most recent echocardiogram completed in March 2021 showed decreased visualization of the bioprosthetic valve vegetation with a stable mitral valve peak velocity of 2.06 m/s.

Recent clinical studies have provided additional insight into Candida IE. One study published by Rivosky et al. monitored 46 patients with prosthetic valve IE who were treated with LAmB versus echinocandin-based induction therapy over a median of nine months. The patients who received only LAmB has a better six-month survival rate compared to patients who received only echinocandins (adjusted odds ratio: 13.52, 95% confidence interval 1.03-838.10) [4]. In addition, 21 patients received long-term maintenance therapy with Fluconazole, with an average of 13 months, and demonstrated minimal adverse effects [4]. Also, the 19 patients who underwent a cardiac surgical procedure did not have improved survival outcomes over a six-month period as compared to the group treated with medical management [4]. These results offer some contradiction to the Infectious Disease of America and European Society of Clinical Microbiology and Infectious Diseases guidelines, which recommend early surgical intervention for all patients with prosthetic IE [5,8]. Despite the poor statistical power with a small sample size, this study provides an insight into medical management as an alternative to surgical therapy.

## Conclusions

Candida IE is a rare pathological process with a high propensity to involve and compromise implanted prosthetic valves and implanted cardiac devices. Individuals with a history of drug use are among the greatest risk in the general population. In our case, the patient had a previous history of bioprosthetic mitral valve replacement and was found to have recurrent IE on his bioprosthetic mitral valve. Ultimately, the patient was not a surgical candidate and was treated with Amphotericin B and long-term Fluconazole. This case focuses on the presentation of *Candida parapsilosis* IE with subsequent imaging findings of this rare disease. In addition, this case highlights the importance of reviewing current clinical guidelines and providing appropriate medical management.
